# Prevention of Anxiety and Depression in Swedish School Children: a Cluster-Randomized Effectiveness Study

**DOI:** 10.1007/s11121-017-0821-1

**Published:** 2017-07-20

**Authors:** Johan Ahlen, Timo Hursti, Lindsey Tanner, Zelal Tokay, Ata Ghaderi

**Affiliations:** 1 Department of Psychology, Uppsala University, Box 1225, 751 42 Uppsala, Sweden; 2 Department of Clinical Neuroscience, Karolinska Institutet, Stockholm, Sweden

**Keywords:** Universal prevention, Anxiety, Depression, School children

## Abstract

Our study aimed at evaluating FRIENDS for Life, an intervention to prevent anxiety and depression in Swedish school children. A total of 695 children between the ages of 8 and 11 were recruited from 17 schools in Stockholm, Sweden, and cluster-randomized to either the intervention or control group. Teachers in the intervention group received a full day of training and administered FRIENDS for Life in their classrooms. We assessed the children’s anxiety and depressive symptoms, general mental health, and academic performance at pre- and post-intervention as well as at the 12-month follow-up. A multi-informant approach was used with data collected from children, parents, and teachers. Assessment was done with the Spence Children’s Anxiety Scale, Children’s Depression Inventory, and the Strengths and Difficulties Questionnaire. Children’s baseline symptoms, gender, and age as well as their teacher’s use of supervision were examined as moderators of effect. Our study found no short- or long-term effects of the intervention for any outcome with regard to the entire sample. We found an enhanced effect of the intervention regarding children with elevated depressive symptoms at baseline. We found a decrease in anxiety symptoms among children whose teachers attended a larger number of supervision sessions, compared to children whose teachers attended fewer supervised sessions or the control group. Mediation analyses showed that this effect was driven by change in the last phase of the intervention, suggesting that supervision might play an important role in enhancing teachers’ ability to administer the intervention effectively.

## Introduction

Already by the age of 16, more than one out of ten children have suffered from an anxiety disorder or depression (Costello et al. 2003). Anxiety and depression are associated with adverse effects in meaningful life areas, including friendships, school performance, and family life, which results in suffering for the child (Birmaher et al. 1996; Donovan and Spence 2000). Moreover, anxiety and depression have been shown to predict future psychiatric diagnoses and increase the risk of suicidal behavior and substance abuse (Bittner et al. 2007; Costello et al. 2003) leading to significant costs for society (Snell et al. 2013). Based on the demonstrably high prevalence, severe consequences for the individual, and high costs to society, it is important to further evaluate methods to prevent anxiety and depression. Especially, since only about 30% of children suffering from anxiety or depressive disorders receive any mental health services (Bienvenu and Ginsburg 2007). Universal prevention is of particular interest, as it potentially involves low costs, does not involve the stigma associated with participation in targeted interventions, and provides an ideal opportunity to access the whole population. However, universal prevention, contrary to targeted interventions, has in general reported small effect sizes, many times not significantly larger than zero (Stice et al. 2009; Teubert and Pinquart 2011). One program of interest, and potentially more effective than average (Fisak et al. 2011), is the widely evaluated Australian program FRIENDS for Life (FFL), a cognitive behavioral prevention program aimed at promoting mental health in children (Barrett 2010). The program developer and her colleagues have conducted three cluster-randomized trials to assess FFL as a universal prevention program. To summarize Barrett’s results, a significantly lower degree of anxiety symptoms has consistently been found in the intervention groups, as compared to the control groups at both post-test and at follow-up. However, the results of depressive symptoms have been somewhat inconsistent between studies, showing both higher and lower depressive symptoms at post and lower depressive symptoms at follow-up. These studies involved interventions administered by teachers (Lowry-Webster et al. 2003), psychologists (Barrett et al. 2006), and teachers or psychologists (Barrett and Turner 2001). In these studies, no differences in effects were found between psychologists or teachers as administrators, which suggests the generalizability and sustainability of the FFL as administered by teachers. Given the possibility of effectively administering the FFL using school staff, the authors argue that FFL may be cost-effective and a good alternative for providing effective prevention to communities with a shortage of trained mental health professionals. However, and in contrast to this optimistic view, more recent studies outside of Australia have not accomplished to replicate these findings when evaluating teacher-administered FFL. Three cluster-randomized trials have been conducted, two in Canada and one in Great Britain. In the two Canadian trials (Miller et al. 2011a, b), school personnel administered the intervention. The results did not indicate a significant difference between intervention and control groups at post- or at follow-up. The trial in Great Britain (Stallard et al. 2014) found significantly lower child-rated anxiety and depressive symptoms in mental health personnel-administered intervention group at the 12-month follow-up compared to the control group. There were no significant differences between the teacher-administered intervention group and the control group. In the previous trials of FFL described above, training and supervision for facilitators have varied between studies. Most studies report an intense 1-day training, but two studies report a 2-day training (Lowry-Webster et al. 2003; Stallard et al. 2014). In contrast to the trials by Miller et al. (2011a, b), the trial conducted by Lowry-Webster et al. (2003) included regular supervision together with the program leader over the course of the 10-week intervention. In the trial by Stallard et al. (2014), teachers were offered supervision every 2 weeks, but the authors report that only a few teachers attended these sessions. In summary, the difference in results between studies of teacher-administered FFL could potentially be partially explained by differences centered in levels of training and supervision of teachers.

Self-ratings of children have generally served as the only outcome measure in earlier trials of FFL, and studies which have included parent ratings have suffered from high incidences of missing data. Also, earlier randomized trials of FFL have in general suffered from inadequate statistical analyses of data, due to a failure to consider clustering effects, which occur within the trials’ designs. In short, not considering clustering effects leads to incorrectly estimated confidence intervals (too small), which implies an increased risk of type I error (Ahlen et al. 2015). Different factors (e.g., age, gender, provider credentials) moderating the effect of preventive interventions have been reported (e.g., Stice et al. 2009; Teubert and Pinquart 2011). However, when only examining universal prevention, these results have not been replicated. Further investigations of factors enhancing the effects of universal prevention program are therefore very important (Ahlen et al. 2015). Our study aimed at evaluating a teacher-administered intervention with multiple informants to provide a comprehensive understanding of the effect of the intervention. Further, our study evaluated whether baseline symptoms, age, gender, and levels of supervision enhanced the effect. The following research questions were addressed: Does a teacher-administered FFL universal prevention program affect (1) children’s ratings of anxiety and depressive symptoms, (2) parent’s ratings of children’s anxiety symptoms and general mental health, (3) teacher’s ratings of children’s emotional problems, pro-social behavior, and academic achievement, and (4) the incidence of anxiety and depressive disorders? Also, (5) do baseline symptoms, gender, age, or teachers’ use of supervision enhance the effect of the intervention?

## Method

### Participants

To find an effect size of Cohen’s *d =* 0.30 in a population with the average correlation between clusters of 0.02 (Ahlen et al. 2015), with a two-tailed significance test (*α =* 0.05), and a power of 80%, it was required to include 18 schools with 35 children from each school (*N* = 630) (Hemming et al. 2011). Our sample consisted of 695 children in third and fourth grade (9 and 10 years old), recruited from 17 public and independent schools in Stockholm County, Sweden. All schools with students of the proposed age range were included, with the exception of very small schools, and all third and fourth graders in these classes were eligible for inclusion. The mean age of the total sample was 9.6 years (*SD =* 0.6). The sample was comprised of 337 girls (48%) and 358 boys (52%). In total, 478 (69%) of the children’s parents contributed with the following demographic information: the parent’s educational level, household income, and country of birth. Table 1 presents demographic characteristics broken down per condition (intervention vs. control).

**Table 1:** Demographic characteristics broken down per condition (intervention and control)

	FRIENDS for Life	Wait list
Child’s age	9.7 years	9.4 years
Gender		
Boys (*n*)	54% (190)	49% (168)
Girls (*n*)	46% (163)	51% (174)
Parent’s educational level		
9-year comprehensive school (*n*)	3% (8)	4% (11)
Upper secondary school, 3 years (*n*)	29% (70)	26% (65)
2-year post-secondary education (*n*)	12% (28)	12% (29)
More than 2-year post-secondary education (*n*)	52% (123)	52% (127)
Graduate studies (*n*)	4% (10)	6% (14)
Median of household income (*n*)	US$6500–7000/month (228)	US$6000–6500/month (235)
Parent’s country of birth		
Sweden (*n*)	75.2% (179)	78.0% (192)
South, East and Southeastern Asia (*n*)	2.7% (7)	3.3% (8)
Middle East (*n*)	7.1% (17)	6.1% (15)
North and East Africa (*n*)	3.8% (9)	2.4% (6)
North and South America (*n*)	2.5% (6)	2.0% (5)
Northwestern Europe (*n*)	2.1% (5)	2.9% (7)
Northeastern Europe (*n*)	1.7% (4)	2.0% (5)
Southeastern Europe (*n*)	4.6% (11)	3.3% (8)

### Procedure

**Fig. 1:**
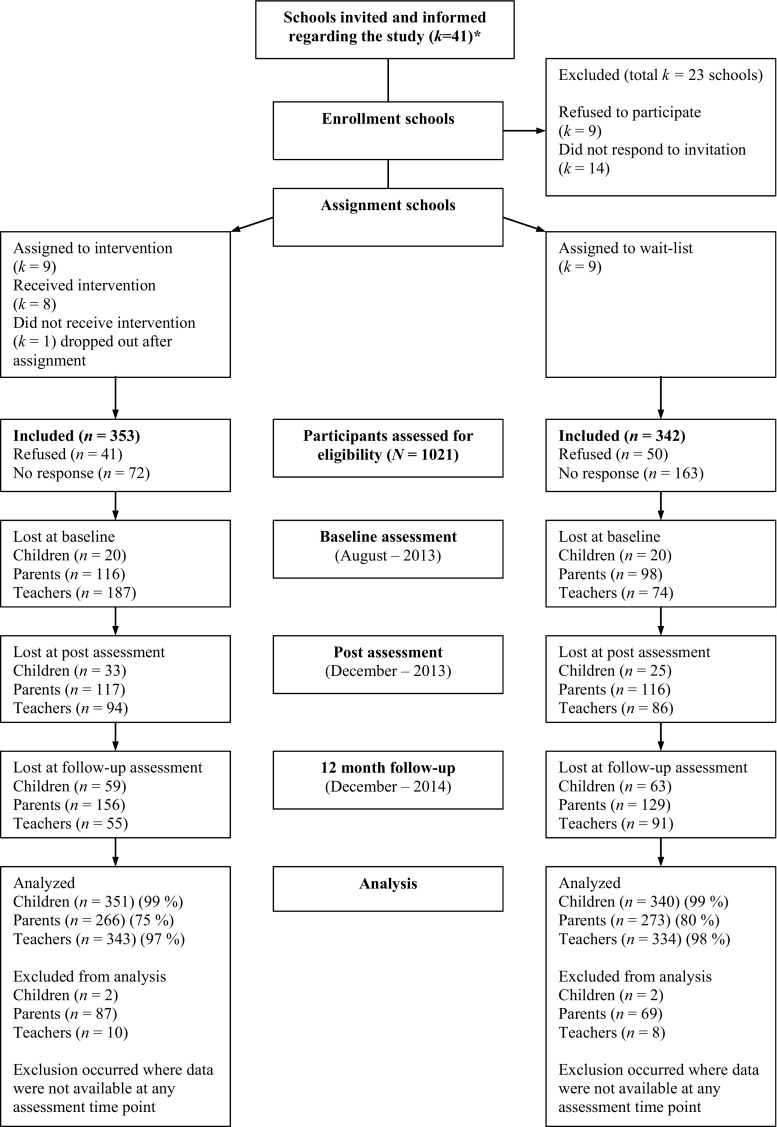
Flow of participants through each stage of the trial

Figure 1 displays a flow of participants through each stage of the trial. As a first step, the research group chose to target six different districts in order to generate a socioeconomically representative sample of Stockholm County and Sweden. A total of 41 urban and suburban schools received information regarding the study and an invitation to participate. Nine schools did not respond, 18 schools declined, thus leaving 18 schools willing to participate. Schools were the unit of randomization. First, schools were ranked based on the educational level of parents. The last author, blind to the schools participating, then generated a random block sequence using Research Randomizer (randomizer.org), containing nine sets of ones and twos, corresponding to intervention and control, respectively. Finally, the block sequence was applied to the list of ranked schools. The randomization of schools took place before the recruitment of participants. This procedure was based on a requirement from several schools, in that the school management needed to know the result of the randomization early, in order to plan the coming semester. One school in the intervention group dropped out after randomization due to engagement in other projects, which resulted in eight schools in the intervention and nine schools in the control group (i.e., 12-month wait-list control). We then sent information regarding the study to all parents of eligible children and asked them to provide written consent in order for their child to participate in the study. Information for parents was translated into seven languages to accommodate for those having a first language other than Swedish. Of the 1021 eligible children, a total of 695 (68%) agreed to participate, 91 (9%) declined, and 235 (23%) did not respond to the invitation.

The trial included three main measurement points: (1) baseline, 1 week before the intervention started; (2) post-intervention, 1 week after the last session of the intervention; and (3) at follow-up, 12 months after the concluding session of the intervention. In addition to these main measurement points, children also completed two intermediate short questionnaires (of anxiety symptoms only) after the fifth and the seventh sessions of the FFL. The children’s questionnaires were completed in the classrooms. All items of the questionnaires were read out loud by the assessor. Parents’ questionnaires were provided via the Internet, and teachers’ ratings were completed during working hours at their convenience. To reduce attrition, additional opportunities to complete questionnaires for children were arranged, and personal e-mails and reminders were sent out to parents. Class teachers in the intervention group administered the intervention 60 min per week, for ten consecutive weeks during school time. Teachers in the control group were instructed to run classes as usual and were asked to not engage in any structured program or educational material during the study period. All teachers who administered the intervention received an intense 1-day standardized training held by the first author, a licensed instructor. The training included information about the early signs of anxiety and depression, risk and protective factors, a rationale for prevention, information on the ethical concerns of implementing FFL, group leadership skills, and a cover-to-cover walkthrough of the FFL group leader manual (Barrett 2010). All teachers facilitating the intervention were scheduled for a supervision meeting after the completion of the third session in the FFL program and were thereafter offered two more supervision sessions during the time of the intervention. The agenda for the supervision sessions included teacher’s description of exercises covered in previous sessions, as well as planning for future sessions, and discussions regarding potential obstacles and difficulties in administering the intervention. During the intervention period, the first author regularly e-mailed and visited teachers at school to make sure they adhered to the intervention schedule. Teachers were offered to record all sessions using a USB recorder. Children in the intervention group also confidentially (at class level) completed a measure of social acceptability at the end of the intervention, including several questions of what they thought about the FFL and how much they learned during the intervention, and additionally one question regarding the degree of homework assignments they received during the intervention. At follow-up, two subgroups (high-anxiety, high-depressive) and a random sample of children not at risk were invited to participate in a structured diagnostic interview. Two master’s level psychology students who were blind to the allocation of schools and hence were able to serve as independent evaluators performed the interviews. These students underwent training, and independently from one another coded three interviews performed by the first author of the current study, to examine inter-rater reliability. The regional ethical review board approved the study, meaning all ethical standards were met.

### Intervention

FFL teaches a number of behavioral and cognitive strategies; all represented by a letter in the FRIENDS acronym. The educational materials include workbooks for children, containing exercises to complete during lessons and homework assignments, and group leader manuals for teachers, outlining objectives and strategies and detailed instructions to all exercises. All educational materials were thoroughly translated and culturally adapted to Swedish children. The first author tried out the FFL material in four school classes, and a focus group of teachers working with children 8–12 years of age carefully examined, and provided feedback on the workbook and group leader manual. The content of the sessions has been comprehensively described by Iizuka et al. (2013) and is therefore only briefly outlined as follows: In the first session, the teacher introduces FFL, and children learn the importance of recognizing and sharing feelings, and being brave. In the second session, the first letter is introduced, F = Feelings. Children learn about different feelings, and how to recognize their own and others’ feelings by looking at facial expressions and body language. In the third session, the second letter is introduced, R = Relax. Children learn to understand bodily signals of different emotions and how different forms of relaxation can help them stay calm and happy. In sessions four and five, the third letter is introduced, I = I can do it. Children learn to identify their self-talk and how helpful and unhelpful thoughts affect our feelings and behavior. In the sixth session, the fourth letter is introduced, E = Explore solutions. Children learn how to overcome problems by dividing problems into smaller steps and practice one step at a time. In the seventh and eighth session, children continue to work on the fourth letter by identifying their social support team and solving problems using a structured problem-solving strategy. In the ninth session, the last letters are introduced, N = Now reward yourself, D = Don’t forget to practice, and S = Smile. Children learn to reward themselves when doing their best and how to use all these strategies in future situations. In the last session, children learn how to maintain the strategies learned in the program.

### Measures

The Spence Children’s Anxiety Scale (SCAS; Spence 1998) is a 44-item child self-report measure of anxiety symptoms (Spence 1998). Essau et al. (2011) found excellent internal consistency of the total scores (*α* = 0.93) in a Swedish sample. Support for convergent and divergent validity were also found; in that, the SCAS showed a significantly larger correlation to the internalizing symptoms of the Strength and Difficulties Questionnaire compared to externalizing symptoms. Internal consistency of total scores in our sample was 0.92. To measure change during the intervention, we used a 12-item short version of the SCAS (SCAS-12; unpublished manuscript). Based on a confirmatory factor analysis of the SCAS modeling six correlated factors, the SCAS-12 was created by choosing two items with the highest loadings from each of the six subscales. Internal consistency of total scores in our sample was 0.85, and the SCAS-12 showed a strong correlation to the total scale, *r* = .95.

The Spence Children’s Anxiety Scale-Parent Version (SCAS-P, Spence 1999) consists of the same items as the SCAS, but formulated from the parent’s perspective. The SCAS-P has not been an object for psychometric evaluation in a Swedish sample. However, in a Dutch sample, Nauta et al. (2004) found good internal consistency of the total scores (*α* = 0.89). Support for convergent and divergent validity were also found, in that the SCAS-P showed a significantly larger correlation to the internalizing compared to the externalizing subscale of the Child Behavior Checklist. Internal consistency of total scores in our sample was 0.88.

The Children’s Depression Inventory-Short Version (CDI-S; Kovacs 2003) is a 10-item rapid version of the original 27-item CDI, a child self-report measure of depressive symptoms. Studies examining the CDI-S have found good internal consistency of the scores (*α* = 0.70–0.80 (Kovacs 2003)). Regarding predictive validity, Allgaier et al. (2012) found the CDI-S to perform as good as the original CDI in diagnostic accuracy. Internal consistency of total scores in the current sample was 0.78.

The Strength and Difficulties Questionnaire (SDQ; Goodman 1997) is a 25-item screening instrument developed to assess children’s mental health. It covers conduct problems, emotional problems, peer problems, hyperactivity-inattention, and pro-social behavior. In a Swedish sample, Smedje et al. (1999) found acceptable internal consistency of the total difficulty and the pro-social behavior scores (*α* = 0.70–0.76), but only fair internal consistency of the emotional problems scores (*α* = 0.61). Support for the convergent validity has been found in that the SDQ correlated highly to the Child Behavior Checklist (Goodman and Scott 1999). Regarding parent ratings, we analyzed total difficulties (SDQ-Tot) and subscales of emotional problems (SDQ-Emo) and pro-social behavior (SDQ-Pro). Teachers only completed the subscales of emotional problems and pro-social behavior. Internal consistency of scores in the current sample were acceptable regarding parent ratings (*α* = 0.70–0.84) and good regarding teacher ratings (*α* = 0.81–0.91).

The Mini International Neuropsychiatric Interview for Children and Adolescents (MINI-KID; Sheehan et al. 1998) is a diagnostic interview for children and adolescents covering several psychiatric disorders. The MINI-KID has shown good to excellent correspondence with another widely used diagnostic interview (i.e., K-SADS-PL), and acceptable to excellent inter-rater and test-retest reliability (Sheehan et al. 2010). Inter-rater reliability was found to be substantial between raters (*κ* = .71).

Additionally, teachers were asked to rate children’s academic performance (AP) according to three questions covering reading, writing, and math skills. Teachers rated children according to a 5-point Likert Scale, much lower than average to much higher than average. We used the mean value for these three questions in the analyses. The internal consistency of scores was good (*α* = 0.88) in the present study sample.

### Data Analysis

We used linear mixed effects regression modeling (LMM) to analyze the effects of the intervention. In repeated measures design, this approach has several advantages to standard repeated measures ANOVA. For example, LMM is able to include higher levels of groups in the analyses (such as schools and classes), covariance structure is modeled to reflect the nature of the repeated observations compared to the standard repeated measures ANOVA where repeated observations are assumed to have the same correlation between each measurement time point. Moreover, in repeated measures LMM, participants with missing observations at some measurement time points could be included in the analysis, which is an important advantage compared to the standard repeated measures ANOVA where participants are deleted listwise (Heck et al. 2013). We performed the LMMs as four-level models with observations nested within subjects, students nested within classes, and classes nested within schools. We specified random intercepts of subjects, classes, and schools. Adding random slopes to the models did not produce significantly better models. We further examined all outcomes for normality, by investigating medians in relation to means, and the magnitude of skewness. Several outcomes were positively skewed. We therefore estimated confidence intervals of the fixed effects using bootstrap procedures as recommended when underlying assumptions of the LMMs are violated (Van der Leeden et al. 2008).

To generate the high-symptom subgroups eligible for participation in the structured interview, we first specified a high-anxiety subgroup by including children with baseline scores more than 1.5 standard deviations above the mean on any of the six subscales of the SCAS. Second, we specified a high-depressive subgroup by including children with baseline scores more than 1.5 standard deviations above the mean on the CDI. A total of 119 children met criteria for the high-anxiety subgroup, and a total of 59 children met criteria for the high-depressive subgroup.

To analyze baseline symptoms, as well as age and gender as possible moderators of the effect, we examined the interaction effects between group and the moderator in a series of LMMs using changes in total scores of the SCAS and the CDI as the dependent variable. We examined short-term effects with pre- to post-change, and long-term effects with change between pre- and follow-up. When examining whether supervision enhanced the effect, we divided the study sample in three groups (1) control group, (2) intervention group with a low rate of supervision (low-supervision group), and (3) intervention group with a high rate of supervision (high-supervision group). Teachers who did not attend, or did only attend the first session of supervision, were categorized as “low rate of supervision.” Teachers who requested and attended to additional supervision were categorized as “high rate of supervision.” See Table 2 for details regarding groups divided by level of supervision. Finally, we also examined possible mediators of change for the different supervision groups. These analyses were performed under the causal inference approach described in detail by for example Imai et al. (2010), as well as Valeri and VanderWeele (2013). In short, the total effect of supervision on the outcome is apportioned into a direct effect (i.e., the outcome is affected directly by the level of supervision, or through other unknown paths), and into an indirect effect, labeled the average causal mediation effect (ACME), which describes the level of supervision’s effect on the outcome driven by mediator levels (Valeri and VanderWeele 2013). The ACME is interpreted as how the outcome on average would change if the mediator level changes from the level expected at low supervision and to the mediator level expected at high supervision, while holding the supervision level constant (Imai et al. 2010). All LMMs were performed in the R software program (R Core Team 2015).

**Table 2:** Number of teachers/classes and group-sizes, together with means and standard deviations of the SCAS broken down per supervision group

Groups	Number of teachers/classes	Class size median (min-max)	Pre (SCAS)		Post (SCAS)		Follow-up (SCAS)	
			M (SD)	*n*	M (SD)	*n*	M (SD)	*n*
High supervision	9	20.5 (18–26)	30.05 (17.65)	134	21.40 (15.76)	137	21.84 (14.04)	124
Low supervision	11	22.5 (15–32)	24.29 (13.84)	199	20.73 (14.64)	183	19.51 (13.05)	170
Control group	22	22.0 (18–27)	27.26 (14.40)	322	21.78 (15.76)	317	20.76 (13.54)	279

## Results

### Attendance, Adherence, and Social Acceptability

The attendance of students was monitored in the intervention group. School class medians of non-attendance ranged between 4.2 and 6.1% between classes. Regarding attendance in supervision, three teachers did not attend the supervision at all, eight attended the first session only, six attended two sessions, and three attended all three sessions offered. Seventeen teachers conducted all ten sessions in the program, two teachers only performed eight sessions, and one teacher six sessions. Unfortunately, only three teachers recorded sessions satisfactorily. Another three teachers participated in recording sessions, but only in small portions. The remaining 14 teachers did not record any sessions. The social acceptability measure was completed by 90% of the children in the intervention group. A total of 80% of the children in the high-supervision group enjoyed FFL “much” or “some” compared to 68% in the low-supervision group. A total of 79% in the high-supervision group thought that they learned much, or quite much about what to do when feeling scared or worried, compared to 69% in the low-supervision group. Furthermore, in the high-supervision group, 33% of classes reported that they had been given homework assignments every week, 44% some weeks, and 22% had not been assigned homework assignments. In the low-supervision group, 9% of classes reported that they had been given homework assignments every week, 9% some weeks, and 82% had not been assigned homework assignments.

### Baseline Comparisons and Attrition Analyses

At baseline, we found a difference regarding age (*t*(690) = 7.27, *p* < .001), where the intervention group was significantly older than the control group (*d =* 0.55). We also found a difference regarding household income (*χ* ^2^(3, *N =* 463) = 10.02, *p = .*02), where the intervention groups had a significantly higher income than the control group (Cramer’s *V* = 0.15). There were also differences regarding teacher’s ratings of the children’s emotional problems and pro-social behavior at baseline (*t*(432) = 5.32, *p* < .001; *t*(432) = 2.11, *p* = .04), where the intervention group had significantly more emotional problems (*d =* 0.54) and fewer pro-social behaviors (*d =* 0.19). Consequently, given it not being a trivial effect size, age was included as a covariate in all analyses, and baseline scores of emotional problems were included as a covariate in teacher ratings of emotional symptoms.

Regarding children who did not complete one or several assessment points, there were no differences in patterns of attrition between intervention or control group. Regarding parents, there was a difference in age, where parents in the intervention group who did not complete measures had older children than parents in the control group who did not complete baseline, post-assessment, and follow-up assessment (*t*(211) = 3.44, *p* < .001; *t*(229) = 3.82, *p* < .001; *t*(281) = 5.06, *p* < .001). Missing teacher ratings appeared to a larger amount in the intervention group at baseline assessment (intervention group, *n =* 187; control group, *n =* 74; *χ* ^2^(1, *N* = 695) = 72.74, *p* < .001). But on the contrary, to a larger amount in the control group at follow-up (intervention group, *n =* 55; control group, *n =* 91; *χ* ^2^(1, *N =* 695) = 12.73, *p* < .001).

### Intervention Effects

Table 3 displays descriptive statistics for all outcomes and measurement points. Effect sizes are presented below as positive when in the desired direction (e.g., when the intervention group showed lower anxiety symptoms than the control group). Two separate repeated measures LMMs showed that there were no significant group*time interactions over the intervention period regarding the child–rated questionnaires the SCAS, *B* = −0.38, 95% CI [−2.48, 1.37], *d =* 0.02, and the CDI-S, *B* = −0.32, 95% CI [−0.71, 0.07], *d =* 0.11. Likewise, four repeated measures LMMs showed that there were no significant group*time interactions over the intervention period regarding the parent-rated questionnaires, the *B* = 0.87, 95% CI [−0.46, 2.31], *d = −*0.03, the SDQ-Tot, *B* = −0.04, 95% CI [−0.72, 0.65], *d =* 0.01, the SDQ-Emo, *B* = −0.07, 95% CI [−0.35, 0.20], *d =* 0.06, or the SDQ-Pro, *B* = −0.14, 95% CI [−0.38, 0.10], *d = −*0.07. Two repeated measures LMMs showed that there were no significant group*time interactions over the intervention period regarding the teacher-rated SDQ-Pro subscale, *B* = −0.27, 95% CI [−0.79, 0.30], *d = −*0.06, or AP, *B* = 0.05, 95% CI [−0.09, 0.19], *d =* 0.12. Finally, a LMM showed no main effect of group at post-assessment regarding the teacher-rated SDQ-Emo subscale, *B* = −0.16, 95% CI [−0.79, 0.49], *d =* 0.04.

**Table 3:** Means, standard deviations, and number of participants for pre-, post-, and follow-up assessments, broken down per condition from raw data

Time-point	Intervention FRIENDS for Life						Wait-list school as usual					
	Pre-		Post-		Follow-up		Pre-		Post-		Follow-up	
	M (SD)	*n*	M (SD)	*n*	M (SD)	*n*	M (SD)	*n*	M (SD)	*n*	M (SD)	*n*
Child ratings												
SCAS	26.60 (15.72)	333	21.02 (15.11)	320	20.49 (13.50)	294	27.26 (14.40)	322	21.78 (15.76)	317	20.76 (13.54)	279
CDI-S	1.77 (2.50)	329	1.72 (2.47)	315	1.55 (2.49)	292	1.82 (2.51)	322	2.02 (3.06)	310	1.63 (2.54)	278
Parent ratings												
SCAS-P	15.45 (9.33)	237	15.06 (10.25)	236	15.35 (10.94)	197	14.6 (9.55)	244	13.00 (8.27)	226	13.92 (10.99)	213
SDQ-total difficulties	7.03 (5.42)	232	7.52 (5.66)	235	7.42 (6.00)	193	6.13 (5.22)	241	6.47 (5.33)	226	6.28 (5.40)	213
Emotional problems	1.68 (1.91)	232	1.61 (1.86)	235	1.72 (2.02)	193	1.24 (1.73)	241	1.23 (1.65)	226	1.29 (1.81)	213
Pro-social behavior	8.38 (1.83)	232	8.19 (1.95)	235	8.23 (1.83)	193	8.50 (1.53)	241	8.43 (1.63)	226	8.43 (1.60)	213
Teacher ratings												
Emotional problems	2.31 (2.61)	166	1.47 (1.96)	259	1.62 (2.30)	298	1.19 (1.75)	268	1.27 (1.86)	256	1.43 (2.08)	251
Pro-social behavior	6.76 (2.71)	166	7.29 (2.76)	259	7.32 (2.78)	298	7.30 (2.50)	268	7.58 (2.30)	256	7.29 (2.80)	251
School performance	3.11 (0.80)	131	3.17 (0.76)	201	3.26 (0.81)	298	3.18 (0.71)	258	3.23 (0.82)	256	3.17 (0.82)	251

Two separate repeated measures LMMs showed that there were no significant group*time interactions over the whole period regarding the SCAS, *B* = −0.07, 95% CI [−1.10, 0.98], *d =* 0.01, and the CDI-S, *B* = −0.09, 95% CI [−0.31, 0.13], *d =* 0.07. Four repeated measures LMMs showed that there were no significant group*time interactions over the whole period regarding SCAS-P, *B* = −0.21, 95% CI [−0.98, 0.55], *d =* 0.04, the SDQ-Tot, *B* = 0.00, 95% CI [−0.38, 0.37], *d =* 0.00, the SDQ-Emo, *B* = −0.07, 95% CI [−0.23, 0.08], *d =* 0.07, or the SDQ-Pro, *B* = −0.07, 95% CI [−.17, 0.10], *d = −*0.04. Further, two LMMs showed that there were no significant group*time interactions over the whole period regarding the teacher-rated SDQ-Pro, *B* = −0.32, 95% CI [−0.35, 0.20], *d = −*0.04, or AP, *B =* 0.07, 95% CI [0.00, 0.13], *d =* 0.15. Finally, a LMM showed no main effect of group at follow-up assessment regarding the teacher-rated SDQ-Emo subscale, *B* = −0.32, 95% CI [−1.38, 0.82], *d =* 0.05.

### Subgroup Analyses

In the high-anxiety subgroup (*n =* 119), we received consent for participation in the MINI-KID for 55 children (46%). Eighteen children (15%) had changed schools, ten children (9%) refused to participate, and 36 (30%) did not respond to the invitation. The participating children did not differ from the non-participating children on any baseline symptom ratings, gender, age, parent’s education, or household income. At 12-month follow-up, 36% of the high-anxiety subgroup in the control condition met criteria for an anxiety disorder, compared to 20% in the intervention condition, *χ* ^2^(1, *N =* 55) = 1.76, *p* = .19. No child met criteria for a depressive disorder at 12-month follow-up; consequently, we did not perform any MINI-KID analyses for the high-depressive subgroup. In the random sample (*n =* 100) of children with no elevated symptoms, we received consent for participation for 50 children, 14 had changed schools, six refused to participate, and 30 did not respond to the invitation. The participating children from the random sample (*n =* 50) did not differ from all other children with non-elevated symptoms (*n =* 501) on any baseline symptom ratings, gender, age, parent’s education, or household income. In the interviewed random sample, 14% of children in the control group met criteria for an anxiety disorder, compared to 9% in the intervention group at 12-month follow-up, *χ* ^2^(1, *N =* 50) = 0.32, *p* = .58.

### Moderation Analyses

A series of LMMs showed no gender*group, or age*group interaction short, or long-term effects for any measure. However, a LMM showed a baseline symptom*group interaction short-term effect regarding the CDI, *B* = 0.39, 95% CI [0.26, 0.53], *d =* 0.43, which implies that higher levels of baseline symptoms involved greater decrease in depressive symptoms between pre and post in the intervention condition (compared to the control condition). In order to in more depth understand the moderation effect of baseline depressive symptoms, we conducted follow-up analyses in three subgroups. These subgroups included children with CDI baseline symptoms (1) above the median (of the current sample), (2) above the third quartile (75th percentile), and (3) above the 90th percentile. Two separate LMMs showed no significant group*time interactions over the intervention period regarding children with baseline scores above the median or the third quartile, *B* = −0.75, 95% CI [−1.63, 0.07], *d =* 0.23 and *B* = 1.00, 95% CI [−2.02, 0.18], *d =* 0.27, respectively. However, a LMM showed a significant group*time interaction over the intervention period regarding children with CDI baseline symptoms above the 90th percentile, *B* = −2.71, 95% CI [−5.12, −0.55], *d =* 0.67. Finally, no significant long-term interaction effect was found regarding the CDI, and no significant short-, or long-term effects were found regarding baseline symptoms*group interaction regarding the SCAS.

### Supervision

There was a significant difference between groups divided by supervision regarding SCAS baseline symptoms, *F*(2689) = 3.279, *p =* .038 and a significant difference in age, *F*(2689) = 27.35, *p* < .001. Consequently, these variables were included as covariates in the analyses. In addition, we examined the supervision groups according to norms presented by the author of the SCAS (Spence 2010a, b). In the high-supervision group, 21 out of 134 children (16%) had elevated levels of anxiety symptoms at baseline assessment. In the low-supervision group and the control group, the corresponding proportions were 16 out of 199 (8%) and 30 out of 322 (9%), respectively. The distribution of children with elevated levels and children without elevated levels of anxiety symptoms was not significantly different between groups, *χ* ^2^(2, *N =* 655) = 5.65, *p* = .06. There was no evidence of problems with outliers (defined as above the T score of 70) in the high-supervision group (*n* = 2, 1.5%), the low-supervision group (*n* = 0), or the control group (*n* = 3, 0.9%). Moreover, these proportions were not significantly different between groups, *χ* ^2^(2, *N =* 655) = 2.59, *p* = .27. A LMM showed a larger short-term (but no long-term) reduction in anxiety symptoms in the high-supervision group compared to the low-supervision group, *B* = 3.27, 95% CI [0.27, 6.15], *d =* 0.22, and the control group, *B* = 2.93, 95% CI [0.11, 5.47], *d =* 0.21. There was no significant difference between the low supervision or control group, *B* = −0.35, 95% CI [2.82, 2.07], *d =* 0.03.

To understand the enhanced effect of the high-supervision group regarding anxiety symptoms, we examined two class-level variables which we hypothesized could be serving as mediators: (1) level of homework assignments and (2) child reports on how much they thought they learned on how to respond to fear or worry. These variables were aggregated values on class level, due to confidentiality on individual level. Furthermore, as a possible individual-level mediator, we additionally examined the intermediate change in anxiety symptoms during the intervention according to the SCAS-12 (i.e., change between sessions 1–5, sessions 5–7, and sessions 7–10), in order to see if the pre-post effect was driven by change in a specific phase of the intervention. Although classes in the high-supervision group had significantly more homework assignments than the low-supervision group (*p* = .02), a mediator analysis showed no significant indirect effect on change in anxiety symptoms (ACME = 0.79, 95% CI [−0.97, 3.05], *p* = .38). There was no significant difference on class averages regarding child reports of what they learned about fear (*p* = .06), and thus as expected, no significant indirect effect on change in anxiety symptoms (ACME = 0.76, 95% CI [−0.79, 2.92, 0.79], *p* = .36). Regarding the individual-level mediator, no indirect effects on change in pre- to post-anxiety was found for the two first phases as mediators (sessions 1–5, ACME = 0.70, 95% CI [−0.34, 1.84], *p* = .16; and sessions 5–7, ACME = −0.27, 95% CI [−1.19, 0.60], *p* = .54). However, an indirect effect was found for the last phase (sessions 7–10) as a mediator, ACME = 0.95, 95% CI [0.05, 2.00], *p* = .04, suggesting that level of supervision increased the reduction of anxiety symptoms at the end of the intervention, which partially explained the difference in pre- to post-changes in anxiety between supervision levels.

## Discussion

The present study aimed at evaluating the effectiveness of the FFL when delivered by classroom teachers to children 8–11 years old in Swedish schools. The results failed to find an effect of the intervention for any outcome regarding the whole population. However, when dividing the intervention group by level of supervision, we found a short-term effect on child-rated anxiety. Further, we also found an enhanced effect on child-rated depressive symptoms for children in the intervention group with elevated depressive symptoms at baseline, suggesting the intervention could be quite meaningful for a subsample of the population. Our study shows similar results as several recent trials of FFL, which have failed to find effects of FFL when implemented as teacher-administered universal prevention (Miller et al. 2011a, b; Stallard et al. 2014). On the contrary, in other trials where FFL has been administered by psychologists or mental health personnel, researchers have found significant effects of the intervention (Essau et al. 2012, Stallard et al. 2014). This is also consistent with the results of recent meta-analyses which have found larger effects of interventions administered by mental health professionals compared to school personnel both regarding anxiety (Teubert and Pinquart 2011) and depression (Stice et al. 2009). A convincing argument to implement a universal intervention in favor of a targeted intervention is the possible cost-effectiveness. Undoubtedly, one way of lowering the costs of an intervention is to let teachers administer it during school hours. However, the optimism that teachers easily can administer the intervention without deflating the effect is seriously put into questioning by our study in resemblance with recent trials. Although similar to recent trials outside of Australia, the results of our study do not harmonize with trials conducted in Australia, where teacher-administered FFL have shown significant effects. There are several possible hypotheses that could explain the disparity in results between studies. One hypothesis is that teachers in different countries may have more or less experience in working with social emotional strategies. Many schools in Sweden have in recent years incorporated the subject “life knowledge” in the curriculum. Even though no teacher in the control group in our study used any comparable program, they may still have incorporated such strategies in their teaching. Second, the creator of the FFL in Australia has continuously developed and improved training, and provided feedback in line with the teachers’ needs. It is possible that the training and supervision of teachers executed in other countries did not reach the same standard were not sufficiently tailored to teachers’ needs or that teachers did not attend to it as scheduled. The analyses based on levels of supervision in our trial lend some support to this hypothesis, as a short-term effect of the intervention was evident among students whose teachers attended a larger number of supervision sessions. The mediation analyses further suggested that this effect was driven by change in the last phase of the intervention. The result of the mediation analysis is theoretically quite plausible and strengthens the evidence that supervision possibly plays an important role in enhancing teachers’ ability to administer the FFL effectively. Basically, teachers in the low-supervision group attended at most one supervision session, which was scheduled after completing the third session. In comparison, the high-supervision group attended additional supervision sessions which was scheduled after sessions five/six, and sessions seven/eight, respectively. Our interpretation of the mediation results posits teachers in the high-supervision group to a larger extent received support in planning the latter sessions, and also better comprehended the strategies learned in these sessions. When interpreting the analyses of levels of supervision, it is important to remember that the effect cannot plainly be interpreted as a treatment effect. Although a reasonable interpretation is that teacher might be able to effectively administer FFL given a larger amount of support, it is also possible that other teacher variables (that covaries with the tendency to attend supervision, e.g., engagement or persistence) drove the pre- to post-changes, rather than the treatment. Moreover, it is also important to underscore that there was no random allocation to levels of supervision. Given the baseline differences in anxiety symptoms between supervision groups, it is possible that teachers with more anxious children in their class were more interested to receive a higher amount of supervision sessions.

### Limitations

One major limitation in the present trial involves the recordings of adherence which did not go according to plan. The recordings of the classroom sessions were technically easily to implement, but the majority of the teachers perceived it as intrusive and refused to record the sessions. The lack of recordings made it impossible to provide a clear and complete account of adherence. Thus, attending the supervision sessions was used as a proxy, which obviously is a limited aspect of the multifaceted nature of adherence. We have assumed that the more the teachers attend the supervision sessions, the more adherent they will deliver the intervention. Although this is also partially reflected in child ratings, we are aware of the difficulties inherent in the nature of this assumption and interpret the outcome cautiously. A continuous collection of recordings would have made us aware of the problems at an early stage and possibly an opportunity to discuss it with the teachers to increase the number of recordings. Further, in addition to the teachers’ low completion rates of recordings and relatively low attendance in supervision, we also encountered some difficulties in collecting parental consent to the structured interviews at follow-up. All in all, these indicators of low engagement highlight the general problem of engaging participants (e.g., teachers and parents) in large longitudinal studies. Low engagement, leading to either attrition or non-compliance or both, obviously involves serious threats to the internal validity of the results. Future trials might benefit from incorporating knowledge generated from implementation research, or even combining effectiveness studies and implementation research as suggested by some researchers (e.g., Curran et al. 2012). Moreover, regarding teachers-ratings, teachers generally rated all children in a class, which meant that attrition appeared in clusters. The results of the teacher ratings should therefore be interpreted with caution, due to the different patterns of attrition between intervention and control group. Finally, the recent meta-analysis by Ahlen et al. (2015) reports very small effect sizes in universal trials regarding anxiety and depression. Following these results, power was a limitation in our study. Specifically, the number of schools might have been too few in order to estimate the standard errors of the effects with adequate precision. Also, having too few randomized units (in our case schools) tends to involve imbalances between the conditions, which in our case was evident especially regarding the mean age in the different conditions. With these limitations in mind, we conclude that if further developed and evaluated as teacher-administered universal prevention in Sweden, efforts should be made to ensure that teachers attend supervision, and adhere the overall implementation of the intervention.
